# The Applications of GIS in the Analysis of the Impacts of Human Activities on South Texas Watersheds

**DOI:** 10.3390/ijerph8062418

**Published:** 2011-06-23

**Authors:** Edmund C. Merem, Sudha Yerramilli, Yaw A. Twumasi, Joan M. Wesley, Bennetta Robinson, Chandra Richardson

**Affiliations:** 1 Department of Urban and Regional Planning, Jackson State University, 3825, Ridgewood Road, P.O. Box 23, Jackson, MS 39211, USA; E-Mails: joan.m.wesley@jsums.edu (J.M.W.); bennrobinson2002@yahoo.com (B.R); CRichardson@suno.edu; (C.R.); 2 National Center for Bio Defense Communications, Jackson State University, Mississippi e-Center @ JSU, 1230 Raymond Road, Jackson, MS 39204, USA; E-Mail: sudha.yerramilli@jsums.edu; 3 Department of Advanced Technologies, School of Agriculture and Applied Sciences, Alcorn State University, 1000 ASU Drive, Jackson, MS 39096, USA; E-Mail: yaw@alcorn.edu

**Keywords:** GIS, watersheds, agricultural watershed, watershed management, South Texas

## Abstract

With water resource planning assuming greater importance in environmental protection efforts, analyzing the health of agricultural watersheds using Geographic Information Systems (GIS) becomes essential for decision-makers in Southern Texas. Within the area, there exist numerous threats from conflicting land uses. These include the conversion of land formerly designated for agricultural purposes to other uses. Despite current efforts, anthropogenic factors are greatly contributing to the degradation of watersheds. Additionally, the activities of waste water facilities located in some of the counties, rising populations, and other socioeconomic variables are negatively impacting the quality of water in the agricultural watersheds. To map the location of these stressors spatially and the extent of their impacts across time, the paper adopts a mix scale method of temporal spatial analysis consisting of simple descriptive statistics. In terms of objectives, this research provides geo-spatial analysis of the effects of human activities on agricultural watersheds in Southern Texas and the factors fuelling the concerns under the purview of watershed management. The results point to growing ecosystem decline across time and a geographic cluster of counties experiencing environmental stress. Accordingly, the emergence of stressors such as rising population, increased use of fertilizer treatments on farm land, discharges of atmospheric pollutants and the large presence of municipal and industrial waste treatment facilities emitting pathogens and pesticides directly into the agricultural watersheds pose a growing threat to the quality of the watershed ecosystem.

## 1. Introduction and Background Information

Over the last several years, the agricultural watersheds in the South Texas region along the coastal plains and the Gulf of Mexico continue to experience repeated problems of impairment and other forms of environmental degradation prompted by human activities and a host of other factors. This is happening in the face of extreme climatic variability coupled with little emphasis on regional assessment and spatial analysis of environmental stressors in the area and its surrounding counties. In view of these voids in the literature, in this paper, we present a Geographic Information Systems (GIS) based analysis of the effects of human pressures on agricultural watersheds, particularly by assessing the ecological and socio-economic issues along the surrounding environment of the South Texas region. According to the literature, recent technological advances have brought GIS techniques to the forefront of management as one of the most viable decision support tools for conservation [1]. In the process, integrated watershed assessment, built on GIS, has in recent times emerged as a recognized system in various nations. It is also proven a vital segment of ecological planning since remote sensing can succinctly track watershed attributes and land use with minor limitations [2]. Ecological modelers have been blending GIS data management and processing ability in the analysis of watershed systems for quite some time. This capability remains indispensable in the design of data infrastructure best suited for watershed analysis [3] and the conservation of natural resources for sustainable development [1,4].

While the current literature largely focuses on issues related to chemical analysis and discharge, water quality in irrigated watersheds, agronomy, forested watersheds, water use efficiency, arsenic concentration and phosphorus management [5–12], the emerging research in areas adjacent to the South Texas basin examined the problem of human impacts on the Rio Grande River through a bio-geographic assessment [13]. The other study that followed a similar vein provided an analysis of the catastrophic effects of human induced changes along stream channels in agricultural watersheds in Illinois [14]. While additional research on the role of land use dynamics in watershed change can be found in Lant’s seminal essay [15], much of the voids in these studies reflect the limited emphasis on the applications of geographic information systems in regional watershed conservation in the coastal plains of the South Texas region for decision-making. Notwithstanding these defects in the literature, the ecological and economic importance of the surrounding watersheds remains quite vital in the life of the region’s population. The region not only contains sensitive natural habitats and water bodies that are essential for biodiversity, but in the last several decades, housing and real estate development, industrial growth, and agricultural activities continue to enhance the revenue base of the area (http://www.fedstats.gov/qf/states/48000.html). Notwithstanding the agricultural watersheds’ role as sources of life support in a complex ecosystem stretching across different counties within the Gulf of Mexico and the coastal plains of Texas, there exists recurrent environmental stress induced by socioeconomic factors.

Accordingly, the environmental concerns in the region consist of air pollution, watershed impairment, and the discharge of waste water containing human pathogens into the water bodies. Other indicators of environmental stress take the form of the growing threats of fecal coli form, widespread use of pesticides in agricultural farming, stream habitat pollution due to the discharge of industrial and agricultural effluents and the menace posed by rapid population growth coupled with the loss of farmland. Institutional response to these problems through different watershed conservation initiatives at the state level remains ad hoc, fragmented, and ineffective in the study area. While the current efforts for managing the water bodies offer opportunities for conservation and best management practices, they are weakened by a lack of regional coordination at the expense of a common strategy towards watershed management and cooperation among the nine individual counties.

In a region prone to numerous ecological disturbances, undertaking watershed based research is often hindered by a lack of access to adequate data infrastructure. Even where data exists for confronting these issues, some of them are incompatible and dispersed in different agencies. This not only widens the geo-spatial data gaps but also hinders efforts in dealing with the monitoring of water problems of the region. At the same time, there exist numerous studies that attest to the applications of geospatial information system in watershed management. In one major study in the literature, Prakash identifies the usefulness of this novel device due to its capability to track fundamental elements shaping the general development and management of a basin, while ensuring minimization of future resource degradation through the adoption of suitable conservation measures [16]. The applications of geospatial analysis in that setting helps managers track the effects of poor stewardship of the environment from extreme anthropogenic activities that trigger ecosystem disturbance in places such as the South Texas region [2].

### Purpose and Objectives and Organization

This paper analyzes the effects of human activities on agricultural watersheds in the South Texas region using Geographic Information Systems (GIS). Emphasis is on the issues, watershed profiles in the study area, efforts to deal with the problem, environmental analysis, factors fuelling the problems and strategies. In terms of methodology, the paper adopts a mix scale approach involving descriptive statistics coupled with geospatial technology. The first aim of the paper focuses on showcasing the use of spatial technology for analyzing human activities on agricultural watersheds while the second objective aims to generate a tool for effective watershed management and conservation. The third objective is to design a decision support tool. The fourth objective is to contribute to the literature. The sections in the paper consist of a review of the issues and trends. Other sections cover the methods and study area profile, the efforts to deal with the problem, ecological analysis of the trends and remedies. The paper concludes with some recommendations.

## 2. Methodology

The study uses primary (original) data in the analysis of the effects of human activities on agricultural watersheds with emphasis on the South Texas region. To map the location of stressors ravaging the watersheds and the extent of their impacts across time and the factors, the paper adopts a mix scale method of temporal spatial analysis consisting of simple descriptive statistics and correlation analysis connected to GIS. In this study, a mix scale approach is referred to as an analysis involving multiple tools such as the integrated use of GIS and simple descriptive statistics. Some of the relevant steps guiding the research consist of a preliminary stage that outlines the identification of variables, data gathering and design as well as data analysis. A detailed description of the steps now follows.

### Stage 1: Identification of Variables, Data Gathering and Design

The initial step guiding the research involves the identification of the 13 variables needed in the research at the county level from 1997–2008. The variables consist of socioeconomic and environmental information made up of acres under farming, harvested cropland, irrigated land, total cropland, acres fertilized, population, market value of agricultural products sold, impaired water bodies, source of pollution, number of waste water facilities draining into watersheds, housing units, ownership rate, building permits, median house hold income (See Tables 1–9). The various categories of data needed for the research were derived from sources such as government documents, newsletters and work found in the libraries. Accordingly, the data gathering process was facilitated by the information provided by the United States Environmental Protection Agency office, the United States Department of Agriculture (USDA), US Census Bureau, the United States Geological Survey (USGS) and the Mississippi Automated Resource Information System (MARIS). That process was followed with the design of data matrices on socioeconomic and environmental variables covering the census periods of 1997 to 2008.

### Identification of Spatial Data Needed For the GIS Mapping

The design of spatial data needed for the GIS analysis required the identification of the appropriate digital county boundary lines covering the study periods of 1997, 2002 and 2007. This entailed the assemblage of the electronic version of available agricultural land resource and land cover maps containing farm producing areas along the watersheds in southern Texas for the periods of 1997, 2002 and 2007. This was made possible through the retrieval of spatial data sets of shape files and grid files from the Mississippi Automated Resource Information System (MARIS) and the USDA in ARCVIEW GIS. Given that the official boundary lines between several counties in the southern part of the state remained stable, it was possible to assign consistent geographic identifier code to the respective area units in order to maintain analytical coherency.

### Stage 2: Data Analysis

**Figure 1.**
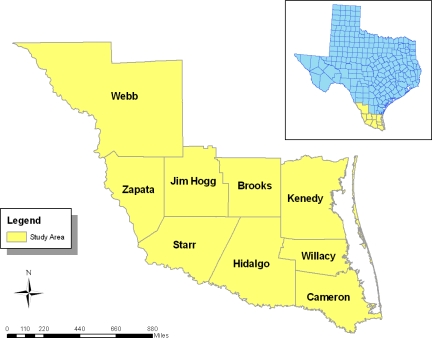
Study area.

In the second stage, agricultural data related to farm operations, and other forms of human activities such as land development and basic descriptive statistics were employed to transform the original data on environmental variables into relative forms of percentages. The purpose of this descriptive statistics analysis is to generate categories and matrices vital to the paper. Under this process, the parameters for establishing the temporal changes in the variables and their relationships to the influence of human activities on agricultural watersheds were generated for the counties in the South Texas region. Emphasizing county level and watershed data analysis across time helps facilitate a gradual appraisal and comparison of the trends in the region over the years. The time series approach is essential in pointing out the changes in the variables from farm land acreage to building permits. This approach allows one to detect levels of change with the tables highlighting the trends and problems facing the study area. The remaining steps of data analysis are somewhat similar to the time series analysis at the county level. They consist of GIS based spatial analysis and output in the form of maps, tables and textual information covering the study periods. The number of spatial units of analysis at the county level consisted of several units as shown in the study area map (Figure 1). The 2007 study area map contains information on several objects such as polygons and lines indicating boundary limits of the counties and their geographic identification codes. The statistical output of the farm land acreage distribution along with those of other variables from the spatial areas units were mapped and compared across time in ARCVIEW GIS. The process helped delineate the spatial locations, patterns and trends highlighting agricultural elements and other socioeconomic and environmental indicators associated with ecological decline along the agricultural watersheds in the study area.

### 2.1. The Study Area

The study area of South Texas as shown in Figure 1 is located in the coastal plains of the state adjacent to the Gulf of Mexico. The South Texas region stretches through a land mass estimated at 8,828.13 square miles made up of nine counties. In 2007, when the study area had a total population of 1,416,930, about 6,476,152 acres of land were devoted to farming with 62,500 acres of farm land treated with fertilizer. As an important region in the state of Texas, the south Texas region not only provides habitat for fisheries but also homes different life forms listed as rare, endangered and threatened [17]. Accordingly, the South Texas Coastal Plain supports unique ecosystems and wildlife, including barrier island dunes, beaches, sea grasses, and the Baffin Bay and Rio-Grande Delta.

**Figure 2.**
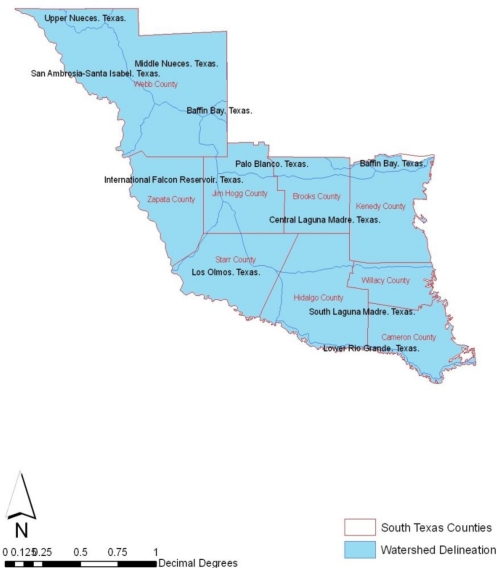
Map of the watersheds.

Some of the essential watersheds in the region include the Baffin Bay, Central Laguna Madre, International Falcon Reservoir, Los Olmos, Lower Rio Grande, Middle Nueces, Palo Blanco, San Ambrosia -Santa Isabel, South Laguna Madre and Upper Neuces (Figure 2). These watersheds are unique because the contributing drainage areas lie in both Texas and Mexico. The composition of the area reflects a mix of urban and rural counties. Among the individual watersheds, the Baffin Bay area is hilly and has varied soil types. Rangeland is the predominant land use in the watershed occupying 63% of the total land area, and the next predominant land use is agriculture, which is 32% of the total. The area’s economy is based on agriculture, petroleum, and tourism. Raising beef cattle is the predominant agricultural activity, although grains, vegetables, cotton and hay are also produced in significant quantity.

In terms of human pressures, the major impacts on this region, over the past 30 years, have been water diversion, flood-control projects, land clearing, pollution, dredging, inter-costal highway construction and pressures from population growth. The Lower Laguna Madre, for instance, has lost about 60 square miles of sea grass cover due to reduced water since the 1960s. Extensive agriculture has fragmented and reduced the areas of native terrestrial ecosystems, and both the Northern and Southern ends of the Padre Island have been developing rapidly as both a resort and residential real estate. In addition, the large number of people now living in “colonials” without sewage treatment not only contribute to the contamination of ground and surface water but also raise a human health problem. This is compounded by un-treated waste water from Mexican municipalities released into the Rio Grande [18]. In a region where 185 waste water treatment facilities drain into the watershed, fecal coli form from ranching and significant quantities of agricultural pesticides and other environmental contaminants are at elevated levels in some of the watersheds [19].

The current evidence of environmental decline in the region does not operate in a vacuum; it may be attributed to human induced socioeconomic factors and stressors. Notable environmental problems threatening agricultural watersheds in the study area consist of environmental stress indicators in the form of declining agricultural land base, population pressure, widespread housing development, growing treatment of farmland with fertilizers, effluents from municipal and industrial waste water and the discharge of pesticides and pathogens into watersheds. The growing threats posed by changing demographics and widespread pollution of the region’s surrounding watersheds and natural environment now makes the use of GIS in analyzing the impacts of human activity on the watersheds more urgent than ever. Under this setting, GIS serves a useful purpose in providing decision support tools for efficient management of the region’s agricultural watershed ecosystem.

### 2.2. The Analysis of Environmental Change

This part of the paper presents environmental analysis with focus on the results of the GIS and descriptive statistics analysis tracking the state of the ecosystem in the area and change over the years from a temporal and geospatial perspective. The analysis of the agricultural land use, fertilizer use, harvested land, total area of cropland, irrigated land, municipal waste discharge and watershed pollution occurring in the area along with the impact assessment of development in the region are also presented. The study area stands as a heavily farmed area with intense activity occurring in all the entire nine counties from 1997 to 2007. This can be buttressed by considering a three tier classification system outlining the distribution of agricultural land among the counties. The first tier county rank based on farmland area during the census years points to Webb county. The farm size in the area as a measure of the level of agricultural activity surpassed the land devoted to farming in other counties listed under the second and third tier categories. With its land base stretching across millions of acres, Webb county stands out in intense farming involving the use of sizable acreages. The second tier counties with land less than a million acres consist of those at Star, Kennedy, Jim Hogg and Hidalgo. The third group of counties with average farmland size measured mostly at less than half a million acres consists of Brooks Zapata, and Cameron. Overall the study area averaged more than a total of 6 million acres in land devoted to farming within much of the study period (Table 1). With a combined total of 1,991,799 acres treated with fertilizers spread through 1997, 2002, and 2007 census years, the study area had an average of nearly 663,933 acres sprayed with fertilizer. A breakdown of the distribution shows an estimated average of over 200,000 acres of fertilized acres at Hidalgo county. The intense fertilizer use there occurred at a level much higher than the other counties. Note also in another group of two counties (Cameron and Willacy), the combined average of fertilized land acreage stood at nearly half a million acres during the 10 year span of 1997 to 2007 (Table 2).

The total size of harvested cropland acreage and the individual values for the respective counties showed only two robust gains in 1997–2002 for Webb and Willacy counties. During the same period, the rest of the study area experienced very significant drops in the size of harvested cropland. With the exception of the drops in Willacy and Hidalgo county, the high rise in total cropland as the data indicates seem to have held steady much of the time. Note also the growing frequency of irrigated land loss that outstretches the soft gains that occurred in just two counties (Tables 3–5).

### 2.3. The Percentages of Change

Over the 10 year period, the study area experienced visible declines in the size of the agricultural land. Between 1997 and 2002, 8 out of 9 counties posted regular declines, while in 2002–2007, only 3 of the nine counties experienced sizable declines. Aside from the trends in the individual counties as shown in the table, the total percentage of change for the area were −9.86%, 9.79 and 0.90%. Over the years the south Texas region experienced visible changes in the use and size of agricultural areas. From 1997 to 2002, 8 of 9 counties posted declines, while in 2002–2007 only 3 of the nine counties experienced substantial drops. The breakdown of the variables showed that while at Brooks county, the rate of change was −5% in 1997–2002 and the size of farm land grew by 19.8%. Elsewhere at both Cameroon and Hidalgo area, the percentages of change were −9.47% to −11.22% during 1997 and 2002, and in the ensuing years of 2002–2007 the size of farmland jumped to 0.27% and 17.9%, respectively. Within the same period, the rate of change in agricultural land in the three counties of Jim Hogg, Kennedy were in the order of −27.7%, −18.36%, and −17.6%. Webb county’s rate of change was estimated at −7.11% in 1997–2002 and dropped further to −10.06% in 2002–2007. Elsewhere, Willacy county posted a gain of +19.85% in 1997 through 2002 but only to drop further to 9.42% from 2002 to 2007. Despite a −5.57% drop in farmland area during the period of 1997–2002, the county gained close to 13.4% between 2002 and 2007 (Table 1).

With some fluctuations, during 1997–2007, fertilizer use in the entire study area rose by 1.30% from 2002 to 2007 (Table 2). On the individual counties, the rate of fertilizer treatment of farmland at Brooks county fell to triple digits values of −155.76%, −112.61% in 1997–2002 and 1997–2007, until it jumped up to 16.87% in 2002–2007. In Cameron county where the size of farm land treated with fertilizer rose by 19.56% and 10.66% in 1997–2002 and 1997–2007, by 2002–2007 the level of fertilizer use in the area fell to −11.06%. In the other counties, Hidalgo County saw the size of its fertilized land area decline by −18.40% in 1997–2002, and by −3.33% in 2002–2007 but only to rebound in 2007 by 12.56%. Within this period, the rate of declines in the application of fertilizer on farm land at Jim Hogg and Kennedy went from moderate to high levels of −24.8%, −45.34%, −81.31% and −43.34% respectively. In both Star and Webb counties where fertilizer use soared over the years, the application rates in the former stood at −6.06% in 1997–2002, but only to move up by 23.07% and 18.41% while in the former, the rate of fertilizer rose by 33.86% to 20.66% until it dropped to −19.95% in 2002–2007. On the remaining areas, while fertilizer applications showed gradual declines of −1.42%, −14.50% and −16.12%, Willacy county’s size of farm land areas treated with fertilizer rose to 74.04% in 2002–2007.

Looking at the harvested cropland table, there is an outright double digit decline in 3 counties with the exception in 2 areas. This left the entire study area with a total decline of −18.46%. With meager decline in total cropland just in two counties, during the study period, there seem to be robust gains in the other counties covered under the census. On irrigated land base, note the mounting decline over the years across the counties. This poses a major problem in the area considering the degree of aridity and lack of rainfall experienced in the area over the years (Tables 3–5).

The pressures unleashed from socioeconomic elements of population and market value of agricultural products sold can be seen with steady rise in total population at over 12% in 2002–2007 and 24.4% in 1997–2007 (Table 6). Aside from a slight drop of −6.42% in 1997–2002, the study area’s market value of tradable farm products increased 32.9% in 2002–2007 and 28.6% in 1997–2007 (Table 7).

### 2.4. Analysis of Impaired Watersheds

In terms of the watersheds in the area, several issues that emerged include the problem of impaired water bodies, percentage of surface waters impaired, source of pollution, number of waste water facilities draining into the water bodies. Of the total of 16 impaired water bodies, the watershed at Baffin Bay and South Laguna Madre were classified under the impaired category while central Laguna Madre had two impaired watersheds. The rest of the water bodies made up of Intercontinental Falcon Reservoir, Los Olmos, Lower Rio Grande, Middle Nueces, San Ambrosio–Santa Isabel and Upper Nueces experienced significant impairment during the period under analysis. On the percentage of surface water area impaired, Baffin Bay, Central Laguna Madre and lower Rio Grande were the three most notable watersheds that each had about 100% of their surface water area impaired. Among others, International Falcons reservoir had 3% of its surface area under impairment while Upper Nueces, San Ambrosio–Santa Isabel and south Laguna Madre had about 13%–17% of their surface water impaired. The sources of pollution on the watersheds as the table indicates consist of pesticides, non-point sources, pathogens, municipal pathogens, and organic enrichments. Another twisting to the status of watersheds in the study area stems from the threats posed by the growing number of waste water facilities draining into the watersheds. From Table 8, note that the watersheds at Baffin Bay and Laguna Madre have 44–47 waste treatment facilities that exceeds the number in the other areas. The number of waste treatment dumping facilities estimated at 17 each in three other watersheds seemed higher than the 11 other operations draining into the central Laguna Madre. Among the other water bodies as the table shows, Middle Neuces had about 13 facilities while Palo Blanco, San Ambrosio–Santa Isable and Upper Neuces maintained 2–7 sites respectively (Table 8).

### 2.5. Spatial Analysis

**Figure 3.**
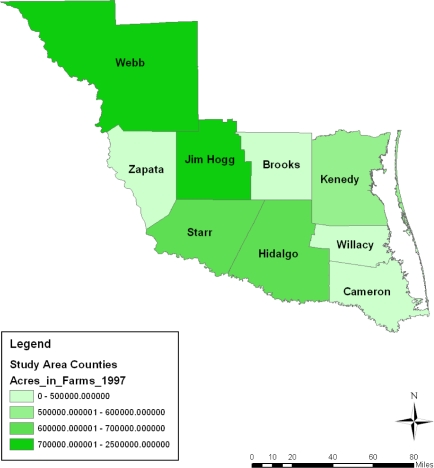
Farm land acreage in 1997.

**Figure 4.**
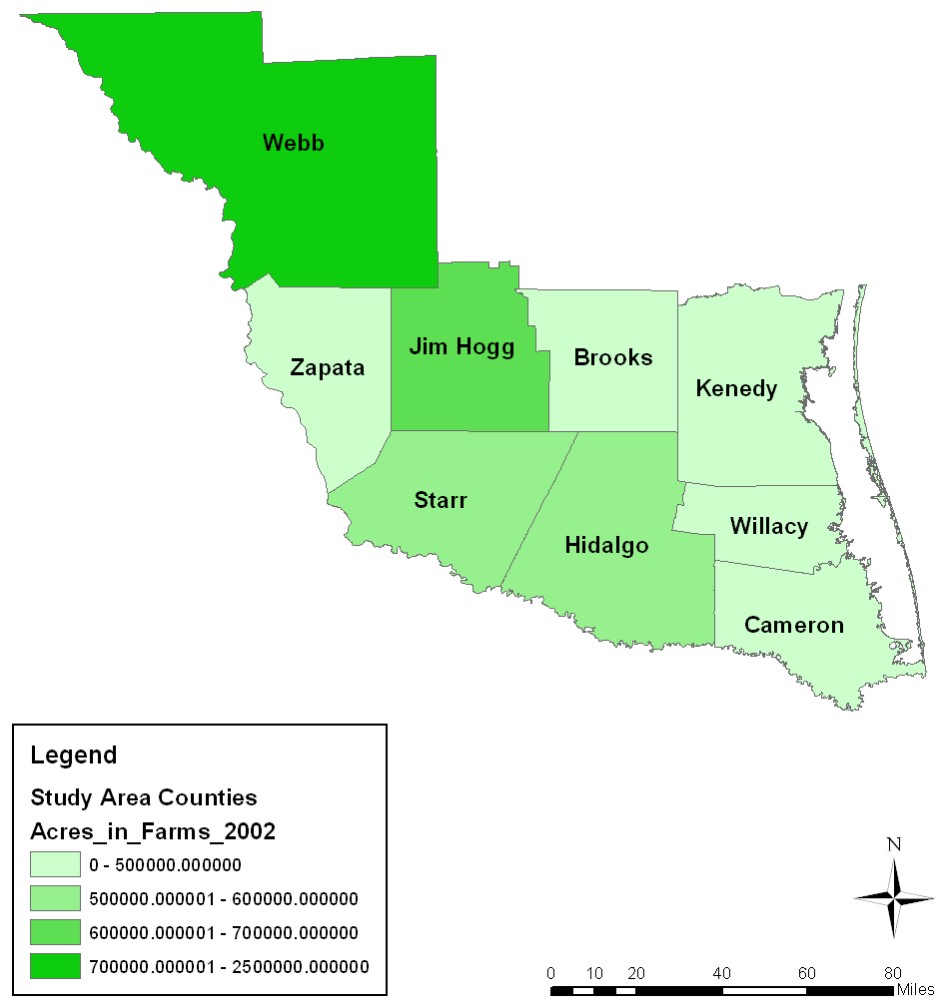
Farm land acreage in 2002.

**Figure 5.**
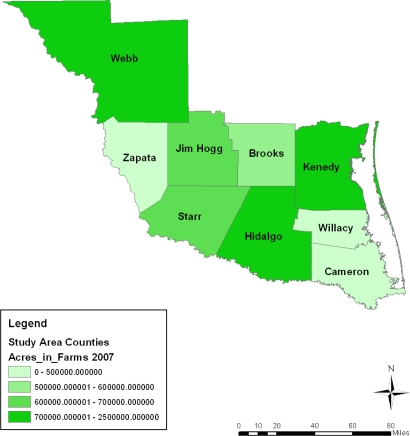
Farm land acreage in 2007.

During the entire period under analysis, the spatial dispersion of the number of farms classified under 10,000–260,000 acres category of land seemed stable in the Webb county portion of the study area for most of the time. The exception to that trend was in 1997 and 2007 when Jim Hogg and the counties of Kennedy and Hidalgo all accounted for a much larger portion of the number of farm operations classified under the 70,000–2,000,000 acres category. The Starr, Hidalgo, and Kennedy counties in 1997 and again the Starr and Brooks counties in 2007 had 500,000–600,000 or 600,000–700,000 acres farm land according to the spatial distribution. Similar patterns reappeared in Starr, Hidalgo and Jim Hogg in 2002. The geographic patterns of land acreage distribution in the lower category of land acreages seemed clustered in the northeast counties of the study area in 2002. They were most notable in the counties of Cameroun, Willacy, and Brooks followed by the North West county of Zapata. Further along the line, comparable patterns resurfaced gradually across space in 2007 and 1997 (Figures 3–5).

**Figure 6.**
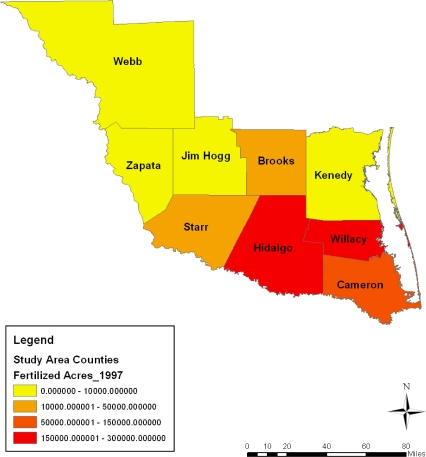
Acreage of fertilized farm land in 1997.

**Figure 7.**
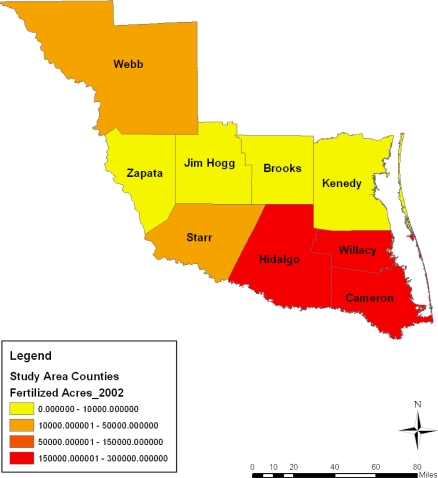
Acreage of fertilized farm land in 2002.

**Figure 8.**
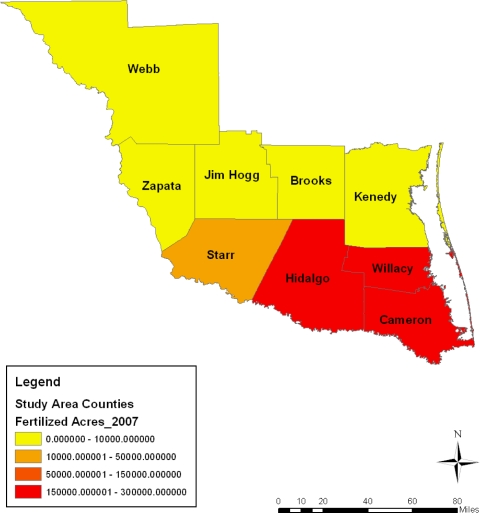
Acreage of fertilized farm land in 2007.

On the spatial distribution of the acreages of farm treated with fertilizers, the south east counties of Cameroun, Willacy and Hidalgo maintained a large concentration of sizable farmland covered with fertilizers at levels much higher than the other areas during 1997, 2002 and 2007. According to the maps, fertilizer treatment on land measuring 160,000–300,000 acres showed more presence most of the time. Just as fertilized areas grouped under the 10,000–50,000 acres category seemed prominent in the counties of Brooks and Starr in 1997, there seems to be a gradual diffusion of similar trends in the Webb county in 2002 as well as a recurrence in Starr county between 2002 and 2007. Considering the large concentration in areas of farm land sprayed with fertilizer under 0–10,000 acres in the northwest portion of the area in 1997, note that by 2002 these spatial patterns not only appeared more along the north central area, but they reemerged in most of the northwest part in 2007 as well (Figures 6–8).

**Figure 9.**
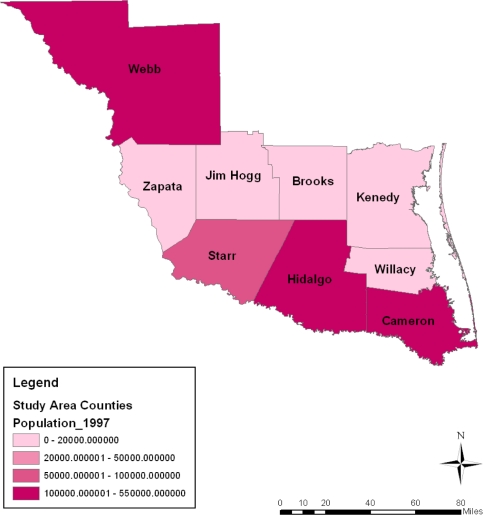
Population map for 1997.

**Figure 10.**
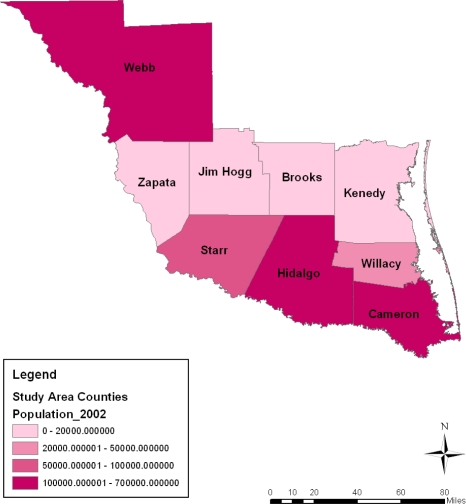
Population map For 2002.

**Figure 11.**
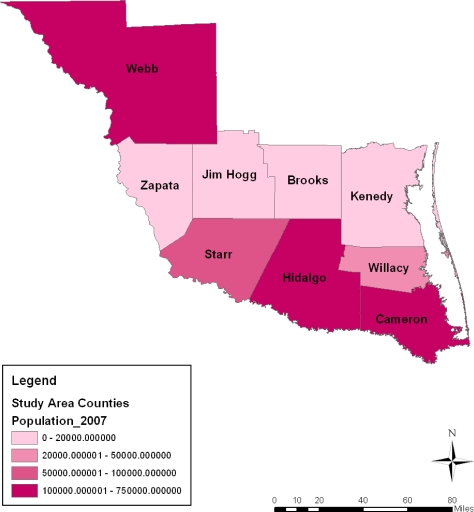
Population map For 2007.

The spatial distribution of population among the counties shows the trends in every category somewhat stable most of the time. Some similarities can be noted between population distribution of every category in 2002 and 2007 followed by a slight difference in 1997. Overall, the breakdown of the figures points to a high population concentration in Cameroon, Hidalgo and Webb counties from 1997 to 2007. During this period, not only did Starr county’s population hold steady (at the 50,000–100,000 category) in the years under analysis, the population concentration across space in the 200,000 category remained visible in the counties of Zapata, Jim Hogg, Brooks and Kennedy (Figures 8–11).

**Figure 12.**
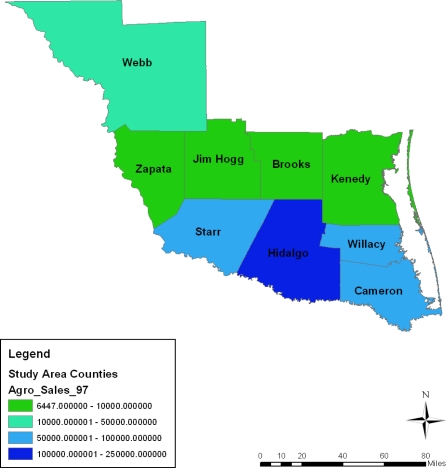
Map showing market value of agricultural products sold ($1000) in 1997.

**Figure 13.**
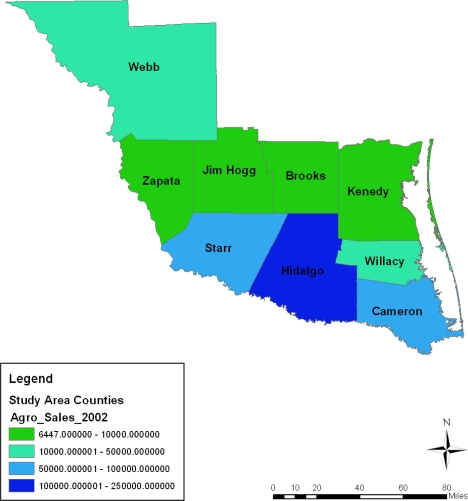
Map showing market value of agricultural products sold ($1000) in 2002.

**Figure 14.**
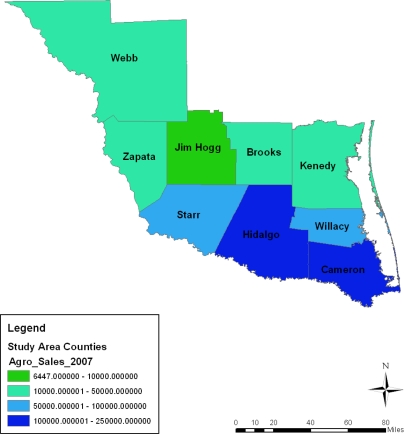
Map showing market value of agricultural products sold ($1000) in 2007.

**Figure 15.**
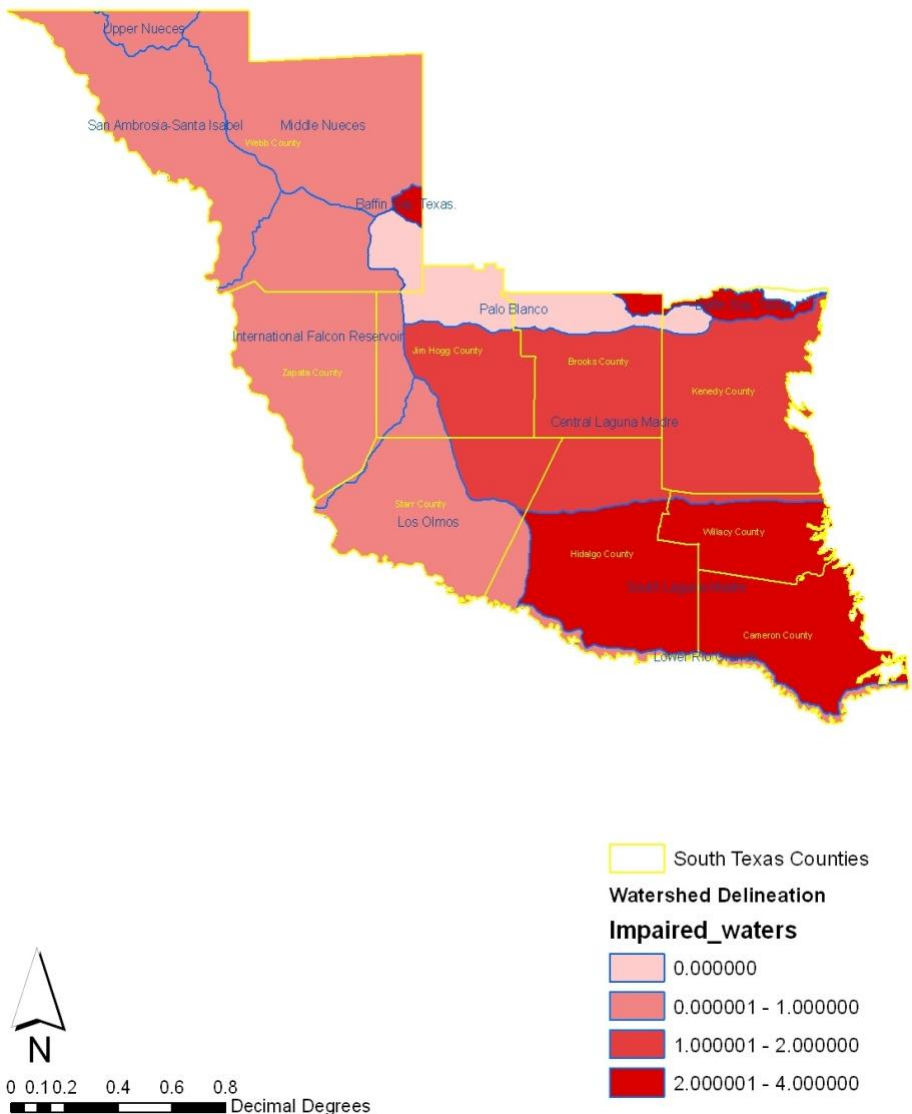
Status of impaired waters in each watershed in the south Texas region in 2007.

In terms of the spatial distribution of market value of agricultural products sold in 1997–2007, the first category of farm products sold under the $1,000,000–250,000 bracket appeared more in Zapata county in 1997, 2002 and 2007 until a gradual diffusion of the trend emerged in Jim Hogg county by 2007. On the geographic dispersion of market value of agricultural products sold in 1997–2007, the initial category of agricultural products sold under the $100,000–250,000 level appeared more in the Zapata County area in 1997–2007. This was followed by a gradual spread of that pattern at Jim Hogg county by 1997. While the second category of income values held steady in Jim Hogg and Starr counties between 1997 and 2002, a gradual cluster of that category emerged at Willacy in 1997 and 2007. In the other years, the lower income class of 10,001–50,000 and 6447–10,000 seemed largely clustered in the southeast and northwest portions of the area (Figures 12–15).

**Figure 16.**
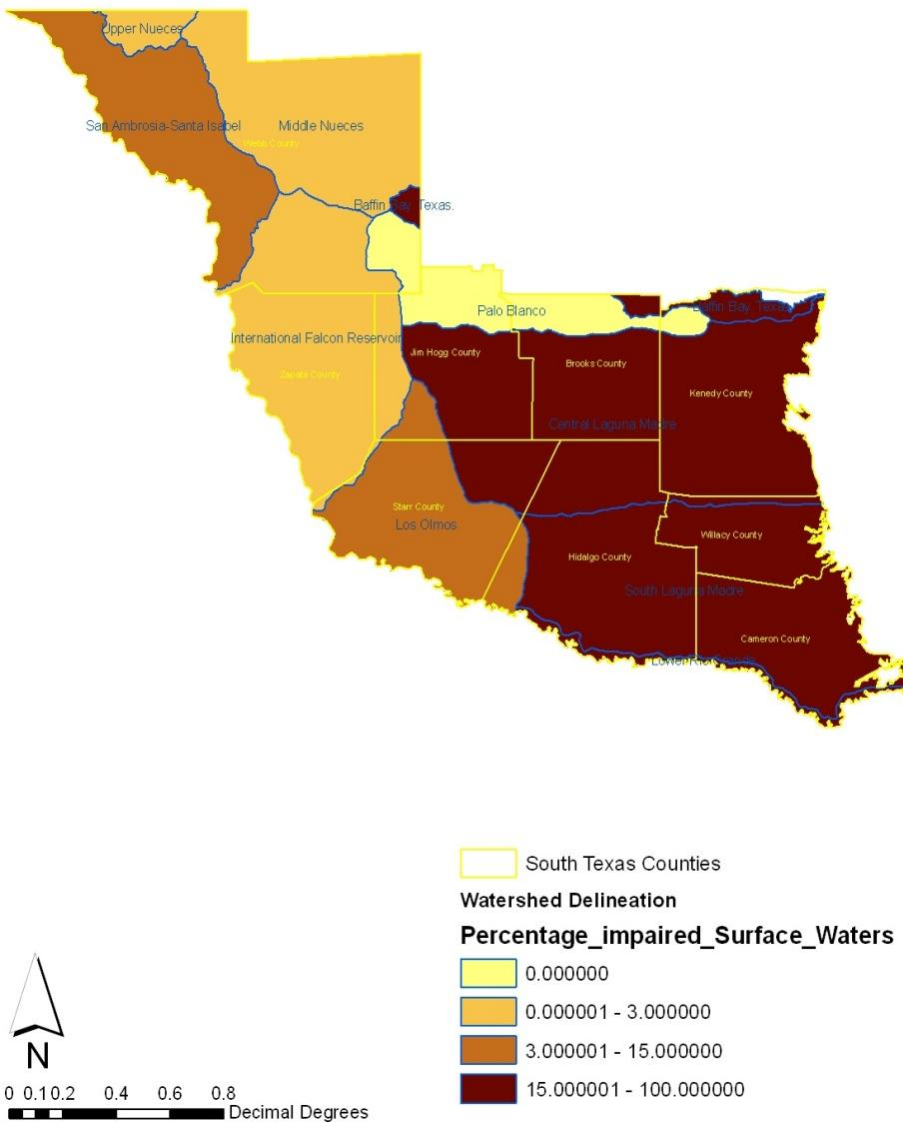
Percentage of impaired surface waters in each watershed in the study region in 2005.

**Figure 17.**
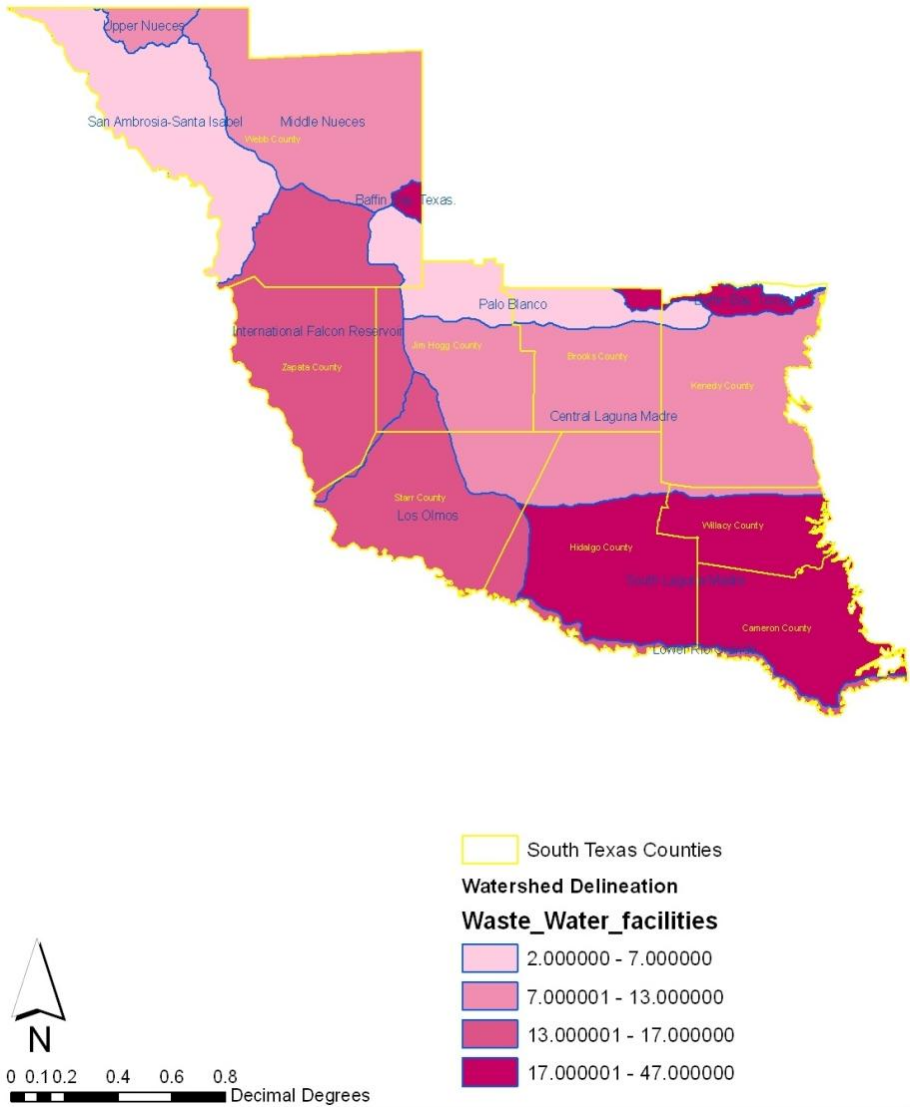
Number of waste water facilities draining pollutants into watershed in 2005.

Turning to the geographic locations of impaired watersheds, number of wastewater facilities, and the percentage of impaired surface waters, the maps show that number of impaired water bodies appear heavily concentrated along the southeast and the central portions of the region in 2007 (Figure 15). Accordingly, the percentage of surface water areas with high level of impairment measuring over 40,000 were not only situated around cities along Hidalgo, Cameroon and Willacy, but impaired water bodies stretching up to 1.00000–200,000 seemed largely spread across the east central counties of Kennedy, Brooks and Jim Hogg. During this period, medium level impairment remained visible in the northeast counties as well. From the map, it is evident that in 2005 most of the watersheds experiencing zero level of impairment maintained a pronounced presence along Palo Blanco (Figure 16). The high number of waste water facilities draining into the watersheds appeared more along the lower end of the southeast portion of the study area. Within the same time in 2005, wastewater treatment facilities classified under the 13000–17000 categories were fully spread across counties in the south west part of the region. More so, treatment facilities of lower capacity appeared largely intense in the north west, the central and upper part of the region (Figure 17).

### 2.6. Factors Responsible for the Problems

The effects of human activities in the region’s watershed does not operate in a vacuum as they are related to several socioeconomic factors, but the analysis herein is limited to only population growth and the market value of agricultural products sold. Among the other areas, the number of residents in the Brooks area stood at 8362, 7806, and 7549 respectively in the periods of 1997, 2002 and 2007. For the Cameron area of the South Texas region, population figures stood at 316,542, 356,745, and 392,736 and in the same period, Hidalgo’s population went from 511,304 in 1997 to over 600,000 in 2002, until it grew further to 726,604 in 2007. Among the low density counties, Jim Hogg saw its opening population figures of 4,929 in 1997 jump to 5,347 during the 2002 period until it dropped to 5,016 in the 2007 period. Kennedy County’s number of inhabitants in the period under analysis was in the order 424, 407 and 388 while Starr county’s population grew from 50,380 to 56,167 from 1997 to 2002 followed by the addition of 62,249 people in 2007. Elsewhere around the high density areas of Webb County, the number of inhabitants jumped from the 1997 estimate of 184,780 to 208,605 in 2002, and in 2007 the population of the area climbed further up to 236,941. Among the midsize counties, Willacy’s population estimated at 19,332 in 1997 rose to 20,288 and 20,600 in 2002 and 2007, respectively. During this period, the population of Zapata county also went from an initial estimate of 10,558 in 1997 to 13,016 and 13,847 in 2002 and 2007 (Table 6).

With the exceptions of Brooks and Kennedy counties where population declines stood at −7.12%, −3.40%, −10.76% and −2.94%, −4.89 and −7.98%, the rise in the number of residents remained quite visible among the rest of the areas. During this period, Cameron County saw its population grow at the rates of 11.26%, 9.15% and 19.40% within 1997–2002, 2002–2007 and 1997–2007 while Hidalgo County experienced notable growth rates of 16.90%, 15.31% and 29.62% respectively. Aside from the population growth rates of 7.81% and 1.73% and the resultant drops of −6.59% at Jim Hogg County, Starr County posted visible increases of 10.30%, 9.77% and 19.06%. During the same periods, Star County had double digit gains estimated at 10.30% to 19.06% along with a 9.77% increase in the number of residents all through 1997–2002, 1997–2007. During this period Webb County also maintained double digit gains of over 11.00% to 21.92%. While the population of Willacy grew at 4.71%, 1.51% and 6.15%, the county of Zapata experienced visible gains of 18.88%, 6.00% and 23.75% respectively.

Among the counties, the economic value of agricultural products sold in the area was in the neighborhood of $8870 for Brooks County in 1997, and in 2002 that number fell to $7573. During the ensuing year of 2007, the value changed to $19,111. Elsewhere Cameroon’s market value of agricultural products sold began with an opening value of $83,365 in 1997 and fell to $74,637 in 2002 only to rebound to $112,350 in 2007. In the same period, Hidalgo county posted an initial market value of farm products of $202,809 in 1997, and this number dropped slightly to $200,073 in 2002. In the following census period of 2007, the figures jumped to $314,256. At Jim Hogg, producers sold farm products worth about $6447, $6940 and $7448 in 1997, 2002 and 2007, respectively. In a similar vein, Kennedy’s market value of farm products sold stood at $6817, $8982 and $18,961 in 1997, 2002, and 2007 respectively. Farmers at Starr county on other hand earned about $51,296 in 1997, $66,744 in 2002 as well as $64,352 in 2007. Of the remaining counties along the South Texas agricultural watersheds, Webb county sold farm products worth $28,078 in 1997, that number changed to $23,639 in 2002 and in the ensuing year of 2007 the value of tradable farm products rose to $24,728. While Willacy county farms sold agricultural items worth $51,131 in 1997, their earnings dropped to $18,907 in 2002 and later grew to $51,200 in 2007. Zapata county on the other hand saw its opening value of $7451 in farm items sold items rise to $9843 and $13,100 in 2002 and 2007, respectively (Table 7).

Between 1997 and 2002, four of the 9 counties in south Texas posted sizable gains in the market value of agricultural products, in the ensuing years of 2002–2007 and 1997–2007 the gains in the value of farm products sold were quite significant in 8 of the 9 counties. Among the individual counties, the market value of farm products sold at Brooks County fell by −17.12% in the opening periods of 1997–2002 and it grew strongly by 60.37% and 53.58% in 2002–2007 and 1997–2007. Just as Cameron county saw its market values of farm products fall to −11.69% in 1997–2007, in the ensuing years, the numbers grew sharply by 33.56% and 25.79%. Notwithstanding the insignificant drop of −0.36% in Hidalgo between 1997 and 2002, the county posted notable gains of over 35% during 2002–2007 and 1997–2007. The rising gains in the market value of farm products were evident in the three counties of Jim Hogg, Kennedy and Starr. In these areas, the percentage of change in the values of farm product sold (between 1997and 2002) stood at 7.10%, 24.10% and 23.14%. These trends continued in a similar fashion as Kennedy county posted highest levels of gains estimated at 52.62% to 64.04% during 2002–2007 and 1997–2007. Furthermore, the level of gains varied from 6.82% to 13.43% at Jim Hogg and 20.28% in Starr county. With a mix of gains and declines in the market value of agricultural products sold in the counties of Webb and Willacy, Zapata county’s market value of farm products sold rose by over 24% to 43.12% during 1997–2002 and the ensuing periods of 2002–2007 and 1997–2007.

Other socioeconomic elements fuelling change in the watersheds can be seen with the rising demands and surge in housing units, growth in home ownership, building permits and income generated through the real estate development. Meeting the needs of a rising population and changing demographics through sale of farm products and housing development puts enough pressure on the environmental resources, as indicated in Table 9. This entails the conversion of arable farmland for residential development, more sewer lines and car usage that eventually results in higher concentration of pollutants along the agricultural watersheds.

### 2.7. Efforts to Deal with the Problems

This part of the paper briefly summarizes the current efforts to mitigate the problems herein analyzed. These initiatives consist of water quality management plan, best management practices by poultry farms, non-point source management program, and watershed plans.

#### Water Quality Management Plan and Best practices

To deal with water quality problems, the Texas Legislature took a major step toward controlling water pollution from agricultural and silvicultural non-point sources when it passed Senate Bill 503 in 1993. Senate Bill 503 authorized the State Soil and Water Conservation Board (TSSWCB) to assist agricultural and silvicultural producers in meeting the state's water quality goals and standards through this voluntary, incentive-based program. With special requirements regarding poultry farms, in the fiscal year 2001, the 77th Legislature amended the Texas Water Code to require all persons who own or operate a poultry facility to implement and maintain a water quality management plan that is certified by the State Soil and Water Conservation Board. The law provides a series of deadlines by which each producer, depending on their initial date of operation, must have requested the development of a WQMP from their local Soil and Water Conservation District. Poultry growers in 45 counties in Texas have received technical and financial assistance from the TSSWCB, through local soil and water conservation districts, to implement practices to abate non-point source pollution from their facilities. Some of those practices include: incinerators, composters, and freezers for mortality management, waste storage facilities for manure management, and nutrient management plans for proper land application of manure as fertilizer.

#### Non-Point Source Management Program

Through a grant program established under Section 319(h) of the Clean Water Act, the U.S. Environmental Protection Agency provides funding to Texas to implement activities that achieve Congress' goal of controlling and abating non-point source pollution. The federal Clean Water Act (CWA) requires states to develop a program to protect the quality of water resources from the adverse effects of non-point source (NPS) water pollution. NPS pollutants include: fertilizers, herbicides and insecticides from agricultural lands and residential areas and bacteria and nutrients from livestock, pet wastes and leaking septic systems. The implementation of the Texas NPS Program involves partnerships among many organizations. With the extent and variety of NPS issues across Texas, cooperation across political boundaries is essential. Many local, regional, state and federal agencies play an integral part in managing NPS pollution, especially at the watershed level. They provide information about local concerns and infrastructure by building support for the kind of pollution controls that are necessary to prevent and reduce NPS pollution. Soil and water conservation districts (SWCDs) are vital partners in working with landowners to implement best management practices (BMPs) that prevent agricultural and silvicultural NPS water pollution.

#### Watershed Protection Plans

A Watershed Protection Plan (WPP) is a coordinated framework for implementing prioritized and integrated water quality protection and restoration strategies driven by environmental objectives. Through the WPP process, the state of Texas encourages stakeholders to holistically address all of the sources and causes of impairments and threats to both surface and ground water resources within a watershed. Developed and implemented through diverse, well-integrated partnerships, a WPP ensures the long-term health of the watershed through strategies to protect unimpaired waters and restore impaired waters. Watershed Protection Plans have a variety of ingredients and can take many forms. The EPA has released a document to help communities, watershed organizations, and local, state, tribal and federal environmental agencies develop and implement watershed plans to meet water quality standards and protect water resources. The handbook is intended to assist individuals undertaking a watershed planning effort. It contains in-depth guidance on quantifying existing pollutant loads, developing estimates of the load reductions required to meet water quality standards, designing effective management measures, and tracking progress once the plan is implemented.

## 3. Results and Discussion

Aside from the state level efforts to alleviate the environmental decline brought about by human activities in the south Texas region, the agricultural watersheds in the area not only remain vulnerable but also still face daunting tasks in containing the ensuing problems of pollution, watershed impairment and the discharge of waste water, fertilizer and pesticide use and the threats of pathogens to the ecosystem. Clearly, the continuous role of human activities and conflicting land uses evident in the region and the ensuing environmental change prompted by various stressors threaten the surrounding ecology of the South Texas region and its watersheds. However, the mix scale analysis involving temporal-spatial techniques of descriptive statistics, connected to Geographic Information Systems (GIS), indicate an increase in human induced stressors of population growth and agricultural land loss. Others include the conversion of agricultural land to other uses, agricultural pollution and other activities. Additionally, the presence of waste water facilities located in some of the counties and rising population and other socioeconomic variables are negatively impacting the quality of water in the agricultural watersheds.

The intense farming activities culminating in the loss of arable agricultural land can be seen with the total size of land under cultivation along the southern region of Texas regions of the water in 1997–2002 and 1997–2007. Aside from minor gains in some counties, analysis of change during the fiscal years show that the use of acreages of land areas and treatment of land with fertilizers seemed rampant in some of the counties with some minor instances of lessened use strong enough to impede ecosystem functions. The same can also be said of harvested areas, total cropland, and irrigated areas. Within the same time, based on data analysis, the population figures in the counties not only stayed strong but also grew for most of the time in 1997–2007, coupled with a slight decrease in a few areas. While the value of agricultural products sold in the market place did rise over the years, the other indicators including number of housing units, demand for building permits, income and host of others showed sizable increase as well.

The implications on the watershed ecosystem health come in the form of prolonged menace of degradation from the widespread use of agrochemicals, the pollution of riparian habitats, and the emergence of other environmental problems. There is also a potential risk of aridity associated with the growing decrease in irrigated land. Furthermore, the continuous rise in population to some degree compounds the declining state of water quality and availability if it is not seriously dealt with. At the same time, enormous financial returns from surplus farm products sold in the market place induces environmental externalities. The greater the increases in market values of traded agricultural products, the higher the use of agrochemicals, pesticides and other nutrients to boost productivity. This not only threatens the carrying capacity of an already fragile watershed ecosystem in the South Texas region, but it poses enormous challenges for both environmental and natural resource managers and policy makers. The incidence of change which is attributed to pressures from demography, economic activities and housing development resulted in more loss of agricultural land, decline in irrigated land, harvested land loss, cropland loss and increased use of fertilizers to boost farm productivity in the areas to meet the needs of inhabitants of the region. The problem is compounded by the threats of waste water treatment facilities and agricultural runoffs.

To map the location of stressors ravaging the watersheds and the extent of their impacts across time and the factors, the paper adopts a mix scale method of temporal spatial analysis consisting of simple descriptive statistics analysis connected to GIS. The design of spatial data needed for the GIS analysis required the identification of the appropriate digital county boundary lines covering the study periods of 1997, 2002 and 2007. With the emergence of GIS and its ability to locate environmental hotspots across time and space, analyzing the spatial diffusion of various ecological stressors known to influence watershed impairment in the South Texas region in various ways serves a useful purpose. This ability remained crucial in the design of data infrastructure best suited for watershed analysis and conservation in the study area. Accordingly, GIS techniques as used here provide a decision support mechanism for managers in the assessment of environmental risks prompted by human activities along watersheds in the 9 area counties of South Texas.

Accordingly, the results of environmental analysis of the trends in the region using a set of ecological, socioeconomic and physical indicators based on temporal spatial assessment using GIS techniques and descriptive statistics analysis found the ecosystem of the South Texas region to be under tremendous stress in some areas due to farming activities and other forms of development. There were also negative environmental impacts from the human activities, including the incidence of pollution caused by the discharge of municipal, industrial and agricultural chemicals into the water bodies. The other negative ecological externalities consist of competing land uses that involved conversion of farmland to other uses, including housing and road construction, to meet the local needs and population increase in the counties. As part of state-wide approach towards mitigation of problems facing the region, the government of Texas did put into place a whole range of measures from water quality management to watershed plan. In the context of the study area, watershed management program based on regional cooperation and GIS analysis of the influence of human activities on the southern Texas region remains quite essential for conservation.

### Recommendations

To deal with the issues identified in the study area, the paper offers five major recommendations for the sustainable use of the watersheds. The suggested areas for future lines of action to boost regional management of the watersheds consist of the need to encourage regional watershed protection planning, development of partnerships among counties, the mitigation of recurrent environmental problems, the adoption of geospatial information system and the development of regional watershed information system.

#### Encourage Coordinated Regional Watershed Protection Planning

Considering the proximity of the counties to each other coupled with the adjoining watersheds, the policy makers in the South Texas region should support coordinated watershed protection planning. This should take the form of locally driven projects that serve as a mechanism for addressing complex water quality problems when water crosses multiple jurisdictions. This will go a long way in protecting the water bodies from impairment and pollution threats. Watershed protection planning serves as a tool for better use of the resources of local governments, state and federal agencies and non-governmental organizations. The planning process involves setting objectives that prioritize implementation projects based upon technical merit and benefits to the community. It also promotes a unified approach that will be useful to the study area.

#### Develop Partnerships among Counties and Other Stakeholders

To deal with the problems facing the nine counties, partnership is a key way to effective watershed management. Through partnership, different people and organizations work together to address common interest and concerns. This partnership can equally involve anyone with a stake in watershed management. Success depends on involving a good list of people and organizations to put together and implement a plan. This can include people with technical expertise, leadership positions, education, public policy, agric-business, and industries, farm organizations, environmental and conservation agencies. Successful partnerships are built on clear and open communication during discussion and outside meetings and should be honest and open. Partners need to listen to each other and provide constructive feedback.

#### Mitigate the Recurrent Environmental Decline along the Watersheds

For years the South Texas basin has been experiencing various kinds of environmental problems that are induced by human activities. The problems include the flow of pollutants and toxic materials in the form of domestic and industrial waste water into water bodies, pesticides, agrochemicals, the loss of farm land, irrigated land decline, and harvested cropland loss. The heavy concentration of pollutants along the water bodies threatens marine life, biodiversity and it overstretches the carrying capacity of the ecosystem beyond limits with serious impacts. Since most of the environmental problems that afflict these watersheds put enormous stress on quality of life and the environment that hinders efforts to confront these issues individually. The paper recommends that concerted efforts be made in these areas in order to mitigate the recurrence of environmental problems in the region.

#### Encourage the Adoption of Geospatial Information System

The analysis in the study area unveiled problems in waste water discharge, population growth, growing use of fertilizers, rises in socioeconomic indicators of income, building permits, market value of traded farm products, and the decline in agricultural land, irrigated land, harvested cropland, and the threats posed to biodiversity. The scale of pressures mounted on the watershed ecosystem in the urban counties reinforces the need for a continuous mapping of the stressors and the use of GIS as a decision support system for policy makers in the region. Accordingly, access to the latest advances in spatial information systems as well as climate risk information offers a sizable advantage to those who have it and hinders the readiness of those who lack it in tackling the problems facing the region. Because the analysis of human impacts on agricultural watersheds is untenable without access to well-designed spatial information systems, the paper recommends that policy makers encourage the use and adoption of these tools for sustainable resource analysis.

#### Develop a Regional Watershed Information System

During the writing of this paper, there existed very little data on the study of watershed management in the South Texas region and as a result, this diminishes the ability of the managers in the region to track and predict impending change jointly. Because decision makers and agencies in the region cannot efficiently manage water resources without periodic access to a central data clearing house, there is a need for the development of a regional agricultural watershed information system as a decision support tool for ensuring the efficient management of resources. The proposed regional data infrastructure should contain information on the physical, socioeconomic and ecological elements influencing watershed impairment.

## 4. Conclusions

This paper has presented the analysis of the impacts of human activities on agricultural watersheds using GIS with a focus on the South Texas region. The paper outlined an overview of the issues in the literature pertaining to the region, the relevance of GIS, the current trends and the state of the ecosystem along the agricultural water bodies in the region. This was followed with the outline of the situation in the South Texas region and its watersheds with some emphasis on the growing threats posed by ecological degradation and widespread presence of environmental stressors. The others include the rise in socioeconomic activities impacting the stability of natural systems, the essence of mix scale approach, the analysis of environmental change indicators and the efforts to mitigate the problems and the factors responsible for the problems.

Having come this far, several significant conclusions can be drawn from this study. Despite the increase from the sale of agricultural products, the use of GIS and descriptive statistics point to a mix of gains and declines in some of the environmental indicators. This trend raises the spectra of responsibilities for planners and those charged with watershed management in the counties of South Texas.

With the emergence of GIS and its ability to locate environmental hotspots across time and space, analyzing the spatial diffusion of ecological stressors known to influence watershed impairment in the South Texas region in various ways will continue to serve a useful purpose. This ability remains crucial in the design of data infrastructure best suited for watershed analysis and conservation. Accordingly, GIS technique as used here provides decision support mechanism for managers in the assessment of environmental risks prompted by human activities along the watersheds in the 9 area counties of South Texas. From the analysis on the South Texas region, the negative environmental impacts ravaging the agricultural watersheds by human actives appear predicated on pressures from demography, the proliferation of urban development and intense farming activities, as well as externalities from municipal and industrial activities in the surrounding ecology of the area. With the increase in fertilizer applications, the rampant use of the river bodies as a sink, the unceasing loss of farmland and the exposure to pollution, the study area faces also the growing threats of human induced stressors.

The absence of joint watershed management initiatives for the South Texas basin region seemed compounded due to the growing threats of stressors concentrating on the boundaries of common water bodies, and meager access to regional data for analyzing the impacts of change. The minimal emphasis on spatial analysis of the state of watershed ecosystem in the area hinders the ability of policy makers to predict the extent and nature of degradation and the ecological costs of human activities. This can be remedied by drawing from the current advances in geospatial information systems in the management of shared waters in the region. This approach remains pertinent as counties in the region grapple with efforts to restore their degraded watersheds. In light of these findings, the practical use of a mix scale approach involving the use of GIS in analyzing environmental change stands as an update to current literature on agricultural watershed management of the South Texas region.

The applications of Geospatial technology as demonstrated in this paper served a vital purpose in providing spatially referenced data for mapping hydrological, socioeconomic and environmental trends. This went a long way in indicating the level of changes in managed waters of the region. The paper also provides the preamble necessary in the design of spatial decision support tools for the management of agricultural watersheds in area.

## Figures and Tables

**Table 1.** Temporal distribution of acres under farm land in the South Texas counties^a^.

**Counties**	**Acres in Farms_1997**	**Farms_2002**	**Farms_2007**	**% Change from 1997 to 2002**	**% Change from 2002 to 2007**	**% Change from 1997 to 2007**
Brooks	465,355	439,771	548,619	−5.81	19.84	15.17
Cameron	383,648	350,437	349,471	−9.47	−0.27	−9.77
Hidalgo	659,696	593,158	722,582	−11.21	17.91	8.70
Jim Hogg	771,228	603,511	640,270	−27.79	5.74	−20.45
Kennedy	561,232	474,073	909,048	−18.38	47.84	38.26
Starr	671,346	570,430	652,780	−17.69	12.61	−2.84
Webb	2,188,035	2,042,680	1,855,894	−7.11	−10.06	−17.89
Willacy	296,333	369,893	338,048	19.88	−9.42	12.33
Zapata	420,941	397,594	459,440	−5.87	13.46	8.37
Total	6,417,814	5,841,547	6,476,152	−9.86	9.79	0.90

a USDA, Census of Agriculture Texas County Data (2007, 2002 and 1997).

**Table 2.** Temporal distribution of acres fertilized in the South Texas counties^a^.

**Counties**	**Acres fertilized_1997**	**Acres Fertilized_2002**	**Acres fertilized_2007**	**% Change from 1997 to 2002**	**% Change from 2002 to 2007**	**% Change from 1997 to 2007**
Brooks	16,420	6,420	7,723	−155.76	16.87	−112.61
Cameron	147,848	183,818	165,501	19.56	−11.06	10.66
Hidalgo	274,367	231,710	265,001	−18.40	12.56	−3.53
Jim Hogg	1,404	1,125	774	−24.8	−45.34	−81.39
Kennedy	No Data	1,160	811	No data	−43.03	No Data
Starr	40,581	38,260	49,739	−6.06	23.07	18.41
Webb	6,742	10,194	8,498	33.86	−19.95	20.66
Willacy	183,707	181,129	158,191	−1.42	−14.50	−16.12
Zapata	No Data	2,200	8,476	No data	74.044	No Data
Total	671,069	656,016	664,714	−2.29	1.30	−0.95

a USDA, Census of Agriculture Texas County Data (2007, 2002, 1997).

**Table 3.** Harvested cropland in the South Texas counties^a^.

**Counties**	**Harvested Cropland_1997**	**Harvested Cropland_2002**	**% Change from 1997 to 2002**
Brooks	No Data	676	No Data
Cameron	112,610	90,188	−19.9%
Hidalgo	189,230	161,402	−14.7%
Jim Hogg	No Data	50	No Data
Kennedy	No Data	No Data	No Data
Starr	8,753	4,887	−44.1%
Webb	1,876	2,582	37.6%
Willacy	17,612	9,325	47.0%
Zapata	No Data	No Data	No Data
Total	330,081	269,110	−18.4%

a USDA, Census of Agriculture Texas County Data (2002 and 1997).

**Table 4.** Total cropland in the South Texas counties^a^.

**Counties**	**Cropland_1997**	**Cropland_2002**	**% Change from 1997 to 2002**
Brooks	64,483	53,611	16.8%
Cameron	229,655	253,571	10.4%
Hidalgo	438,908	405,094	−7.7%
Jim Hogg	25,078	42,798	70.6%
Kennedy	No Data	6,289	No Data
Starr	126,566	193,688	53.0%
Webb	51,629	90,036	74.3%
Willacy	234,279	230,450	−1.63
Zapata	32,605	63,819	95.7%
Total	1,203,203	1,339,356	11.3%

a USDA, Census of Agriculture Texas County Data (2002 and 1997).

**Table 5.** Irrigated land in the South Texas counties^a^.

**Counties**	**Irrigated Land_1997**	**Irrigated Land_2002**	**% Change from 1997–2002**
Brooks	1,190	811	−31.8
Cameron	117,579	104,770	−10.8
Hidalgo	195,086	170,284	−46. 2
Jim Hogg	No Data	2,386	No Data
Kennedy	No Data	No Data	No Data
Starr	10,606	6,133	−42.1
Webb	5,985	7,101	18.6
Willacy	18,451	10,390	−43.6
Zapata	1,725	2,994	73.5
Total	350,622	304,869	−13.0

a USDA, Census of Agriculture Texas County Data (2002 and 1997).

**Table 6.** Population statistics in the South Texas counties^a^.

**Counties**	**Pop_1997**	**Pop_2002**	**Population_2007**	**% Change in 1997–2002**	**% Change in 2002–2007**	**% Change in 1997–2007**
Brooks	8,362	7,806	7,549	−7.12	−3.40	−10.76
Cameron	316,542	356,745	392,736	11.26	9.16	19.40
Hidalgo	511,324	615,343	726,604	16.90	15.31	29.62
Jim Hogg	4,929	5,347	5,016	7.81	−6.59	1.73
Kennedy	419	407	388	−2.94	−4.89	−7.98
Starr	50,380	56,167	62,249	10.30	9.77	19.06
Webb	184,980	208,605	236,941	11.32	11.95	21.92
Willacy	19,332	20,288	20,600	4.71	1.51	6.15
Zapata	10,558	13,016	13,847	18.88	6.00	23.75
Total	1,106,826	1,283,724	1,465,930	13.78	12.42	24.49

a US Bureau of Census (2007, 2002, 1997).

**Table 7.** Market value of Agricultural products sold in the South Texas counties^a^.

**Counties**	**Market value_1997**	**2002**	**2007**	**% Change from 1997–2002**	**% Change from 2002–2007**	**% Change from 1997–2007**
Brooks	8,870	7,573	19,111	−17.12	60.37	53.58
Cameron	83,365	74,637	112,350	−11.69	33.56	25.79
Hidalgo	202,809	202,073	314,256	−0.36	35.69	35.46
Jim Hogg	6,447	6,940	7,448	7.10	6.82	13.43
Kennedy	6,817	8,982	18,961	24.10	52.62	64.04
Starr	51,296	66,744	64,352	23.14	−3.71	20.28
Webb	28,078	23,639	24,728	−18.77	4.40	−13.54
Willacy	51,131	18,907	51,200	−170.4	63.07	0.13
Zapata	7,451	9,843	13,100	24.30	24.86	43.12
Total	446,264	419,338	625,506	−6.42	32.96	28.65

a USDA, Census of Agriculture Texas County Data (2007, 2002, 1997).

**Table 8.** Status of watersheds in the study region^a^.

**Watershed**	**Impaired Water bodies**	**Percentage of surface waters impaired**	**Source of pollution**	**Number of waste water facilities draining into watershed**
Baffin Bay	4	100	Pesticides	44
Central Laguna Madre	2	100	Non-point sources	11
International Falcon Reservoir	1	3	Pathogens	17
Los Olmos	1	15	Municipal Point sources	17
Lower Rio Grande	1	100	Pathogens	17
Middle Nueces	1	3	Pathogens	13
Palo Blanco	0	0	Pathogens	2
San Ambrosia–Santa Isabel	1	13	Municipal Point sources	7
South Laguna Madre	4	76	Pathogens/pesticides/organic enrichment	47
Upper Nueces	1	2	Pathogens	10

a USEPA, Watershed Assessment, Tracking & Environmental Results, 2002.

**Table 9.** Socioeconomic statistics in the South Texas counties^a^.

**Counties**	**Housing Units_2008**	**Owner-Occupied Housing Units**	**Renter-Occupied Housing Units**	**Homeownership Rate_2000 (%)**	**Median Household Income_2007**	**Building Permits_2008**
Brooks	3,269	5,629	2,275	73	24,208	6
Cameron	145,625	65,875	31,392	67.7	29,589	1,306
Hidalgo	253,366	114,580	42,244	73.1	31,353	3,694
Jim Hogg	2,402	1,409	406	77.6	32,350	0
Kenedy	292	48	90	34.8	30,581	0
Starr	18,372	11,450	2,960	79.5	23,070	0
Webb	70,702	33,322	17,418	65.7	33,697	1,087
Willacy	7,202	4,316	1,268	77.3	24,961	34
Zapata	6,506	3,212	709	81.9	30,017	0

a U.S. Census Bureau (2009): Fedstats Map Stats Texas.
